# Protein Intake among Community-Dwelling Older Adults: The Influence of (Pre-) Motivational Determinants

**DOI:** 10.3390/nu14020293

**Published:** 2022-01-11

**Authors:** Marije H. Verwijs, Annemien Haveman-Nies, Jos W. Borkent, Joost O. Linschooten, Annet J. C. Roodenburg, Lisette C. P. G. M. de Groot, Marian A. E. de van der Schueren

**Affiliations:** 1 Department of Nutrition, Dietetics and Lifestyle, School of Allied Health, HAN University of Applied Sciences, Kapittelweg 33, 6525 EN Nijmegen, The Netherlands; m.h.verwijs@hva.nl (M.H.V.); jos.borkent@han.nl (J.W.B.); 2 Division of Human Nutrition and Health, Wageningen University & Research, Stippeneng 4, 6708 WE Wageningen, The Netherlands; lisette.degroot@wur.nl; 3 Department of Social Sciences, Consumption and Healthy Lifestyles, Wageningen University & Research, Hollandseweg 1, 6706 KN Wageningen, The Netherlands; annemien.haveman@wur.nl; 4 Department of Food Science & Technology, HAS University of Applied Sciences, P.O. Box 90108, 5200 MA Den Bosch, The Netherlands; J.Linschooten@has.nl (J.O.L.); A.Roodenburg@has.nl (A.J.C.R.)

**Keywords:** aged, proteins, diet, behavior

## Abstract

An adequate protein intake is important for healthy ageing, yet nearly 50% of Dutch community-dwelling older adults do not meet protein recommendations. This study explores protein intake in relation to eight behavioral determinants (I-Change model) among Dutch community-dwelling older adults. Data were collected through an online questionnaire from October 2019–October 2020. Protein intake was assessed by the Protein Screener 55+, indicating a high/low chance of a low protein intake (<1.0 g/kg body weight/day). The behavioral determinants of cognizance, knowledge, risk perception, perceived cues, attitude, social support, self-efficacy and intention were assessed by evaluating statements on a 7-point Likert scale. A total of 824 Dutch community-dwelling older adults were included, recruited via online newsletters, newspapers and by personal approach. Poisson regression was performed to calculate quartile-based prevalence ratios (PRs). Almost 40% of 824 respondents had a high chance of a low protein intake. Univariate analyses indicated that lower scores for all different behavioral determinants were associated with a higher chance of a low protein intake. Independent associations were observed for knowledge (Q4 OR = 0.71) and social support (Q4 OR = 0.71). Results of this study can be used in future interventions aiming to increase protein intake in which focus should lie on increasing knowledge and social support.

## 1. Introduction

Protein-energy malnutrition (PEM) affects 7 to 12% of Dutch community-dwelling older adults [1]. PEM, in combination with reduced physical activity, may lead to physical limitations, such as the inability to walk stairs or go grocery shopping [2]. It is known that Dutch older adults are often unaware of the importance of an adequate protein intake and of problems associated with malnutrition [3,4,5]. To ensure that the increasing number of older adults [6] remains vital and independent as long as possible, an adequate intake of dietary protein is crucial [7,8]. In contrast to WHO recommendations (0.8 g of protein per kg body weight per day (g/kg BW/day) [9], the European Society for Clinical Nutrition and Metabolism recommends an intake of 1.0 g protein/kg/day [10]. Approximately 50% of Dutch community-dwelling older adults do not meet this recommendation [11] and especially during breakfast, protein intake is too low in this population [12]. To counteract this low protein intake, more insight into protein-related dietary behavior is needed to look for opportunities for sustainable behavior changes for the majority of this population.

Dietary behavior is influenced by various determinants. Behavioral change theories have been developed describing multiple factors that may explain dietary behavior [13]. One of those theories is the I-Change model [14,15,16]. This model distinguishes three phases prior to actual behavior: awareness, motivation and action. All three phases incorporate four behavioral determinants that are relevant to the corresponding phase, and that can eventually influence behavior. “Awareness” consists of the behavioral determinants: “cognizance, knowledge, risk perception and perceived cues; motivation comprises attitude, social support, self-efficacy and intention while action planning, plan enactment, skills and barriers” are behavioral determinants within the “action” phase. 

Awareness about the importance of an adequate protein intake and the problems associated with malnutrition is low among older adults [3,4,5]. Therefore, older adults are likely to be in the “awareness” phase of the I-Change model, rather than the phase in which taking action is considered. However, it is also relevant to assess motivational factors with regard to protein intake, especially since about half of older adults meet protein recommendations. Until now, little research has been performed concerning behavioral determinants that may influence protein intake in Dutch older adults. Therefore, this study examines the association between the chance of a low protein intake and eight behavioral determinants within the “awareness and motivational” phase of the I-Change model among Dutch community-dwelling older adults. 

## 2. Materials and Methods

### 2.1. Study Design and Sampling

This study was a cross-sectional study in which data were collected through an online questionnaire administered from October 2019–October 2020. Inclusion criteria were as follows: community-dwelling older adults of 65 years and older (with or without home care); and being able to complete an online questionnaire (independently or with help). A total of 824 respondents were recruited through multiple strategies: online newsletters of a Dutch association for older adults (*n* = 201), a newsletter of a health insurance company (*n* = 229); a video in a national newspaper and various articles in regional newspapers across the Netherlands (*n* = 296), and through personal approach (*n* = 98). 

The questionnaire had been piloted for readability and comprehensibility among twelve older adults, recruited through personal approach. The results of this pilot were used to finalize the questionnaire.

### 2.2. Measurements

The questionnaire consisted of 50 items and started with a short description of the aim of the study and the content of the questionnaire.

### 2.3. Socio-Demographic Characteristics

The first 14 items asked for socio-demographic characteristics: age, gender, marital status, living situation, living area, having children (yes/no), country of birth, type of home care (if relevant), educational level, income, self-reported body weight, body height and weight loss during the past three months. Respondents with implausible weight and height (<40.0 kg and <1.00 m) were excluded from the data set (*n* = 8). 

### 2.4. Protein Intake

Protein intake was assessed using the Protein Screener 55+ (Pro55+). The outcome of this screening tool provides an indirect measurement of protein intake as it estimates the chance of a low protein intake through a prediction model, the so-called probability score. A protein intake below 1.0 g/kg BW/day is considered a low protein intake. This screening tool has been validated among Dutch older adults aged ≥55 years against outcomes of a food frequency questionnaire (FFQ) in a large population of Dutch participants (*n* = 5188) and is considered to be a valid method to assess low protein intake in this population, based on an area under the curve (AUC) of 0.856 in the validation sample [17]. Based on the validation study [17], a cut-off value for the probability score of 0.3 was used: a score higher than 0.3 indicates a high chance of a low protein intake (<1.0 g/kg BW/day) while a score below 0.3 indicates a low chance of a low protein intake. The probability score is based on body weight and results of ten items regarding consumption frequency and portion size of ten protein-rich food items. Examples of food items are “In the last 4 weeks, how often did you eat eggs with either your breakfast, lunch, evening meal, as a snack, or in a meal?” or “In the last 4 weeks, how many slices of bread did you eat on an average day?”. Participants were asked to indicate the frequency in “Not in these 4 weeks” to “7 days/week” and the amount in “None/Less than one, or not applicable” to “More than 12”. 

### 2.5. Behavioral Determinants

The questions regarding behavioral determinants “cognizance, knowledge, risk perception and perceived cues, attitude, social support, self-efficacy and intention” followed the Pro55+. In the question that assessed the level of “knowledge” concerning protein-rich food items, respondents were asked to indicate which of the listed foods contain protein. Twelve types of food were shown through pictures (bread, eggs, fruit, vegetables, marmalade, coffee, milk, nuts, olive oil, fruit juice, meat and salmon). The behavioral determinant “knowledge” was assessed by scoring: +1 for correct answers, −1 for incorrect answers. This led to a minimum score of −6 and a maximum score of +6. 

The other items covered the seven other determinants of behavior concerning protein consumption: “cognizance, risk perception, perceived cues, attitude, social influences, self-efficacy and intention”. Since protein intake is known to be especially low during breakfast, questions were formulated as “throughout the day” and/or specifically “during breakfast”. Even though it was intended to analyze the outcomes per timing of the day, outcomes of Spearman’s rho correlation were high (>0.50) in most behavioral determinants. This indicates that there were only small differences in outcomes per timing of the day.

The questions were based on existing questionnaires and literature describing how to identify specific behavioral determinants [14,18,19,20,21,22,23]. Table 1 gives an overview of the items regarding the behavioral determinants. For every behavioral determinant, two-to-six items were incorporated into the questionnaire. Respondents were asked to rate to the extent they agreed with the statements on a 7-point Likert scale (totally disagree–totally agree). An average per behavioral determinant was calculated to obtain an overall score of each determinant. 

### 2.6. Procedure

After answering the items regarding the socio-demographic characteristics, Pro55+ and the determinant “knowledge”, respondents received information on products that naturally contain dietary protein to support appropriate answering to questions on the remaining seven determinants. The questionnaire ended with a thank you note including the possibility to download the information that was given during the questionnaire and links to websites with extra information. 

### 2.7. Internal Validation

Cronbachs α of seven behavioral determinants (except “knowledge” as this was only one question) were calculated to check whether the separate items per behavioral determinant could be combined into one scale (Table 1). A Cronbachs α ≥ 0.70 is viewed as an acceptable value for internal consistency [24]. For “cognizance” and “self-efficacy” Cronbachs α was below 0.70. Therefore, sensitivity analyses were performed (Appendix A; Table A1) by analyzing the separate items of “cognizance” and “self-efficacy” as well as the combined scales as described in the section “behavioral determinants”. As the separate items of the scale showed outcomes relatively comparable to the combined scale, we decided to use the combined scale for “cognizance” and “self-efficacy” instead of the separate items despite the low Cronbachs α.

### 2.8. Ethical Considerations and Data Management

The HAN Ethical Advisory Board judged the study protocol and concluded that this study did not fall within the remit of the Medical Research Involving Human Subjects Act (WMO). Respondents received detailed information about the aim, content, and data storage of the study before the start. By accepting the terms of agreement, informed consent was signed. Respondents were able to discontinue completing the questionnaire at any time. Data were stored confidentially. 

### 2.9. Data Analysis

The answers to the different questions for the behavioral determinants were not evenly distributed over the seven-point Likert scale and only few respondents chose the first three categories. Therefore, quartiles were composed based on the outcomes of the behavioral determinants. As logistic regressions provide an overestimate of the prevalence (ratio’s), Poisson regressions with robust variance estimations were used to calculate prevalence ratios [25]. In these analyses, protein score (high vs. low chance of a low protein intake) was included as the dependent variable and knowledge and the seven behavioral determinants as independent variables. Prevalence ratios (PR) were reported with corresponding 95% confidence intervals (model 0) and additionally adjusted for age (65–74 years; 75–84 years; >85 years), gender (male/female), BMI (<20 kg/m^2^; 20–27 kg/m^2^; >27 kg/m^2^), living situation (alone/together) and income (low/high); model 1. A full model was developed in which all behavioral determinants were included (model 2). For the full model, variance inflation factor (VIF) scores were assessed in R version 4.0.2 (car package) to test for multicollinearity. VIF scores for all variables were <5, indicating no multicollinearity.

## 3. Results

Sociodemographic characteristics of the respondents (*n* = 824) are shown in Table 2. Of all participants, 39.4% had a high chance of a low protein intake (<1.0 g/kg BW/day). There were only slight differences in sociodemographic characteristics between respondents with a low and high chance of a low protein intake. Mean age was 72.9 years, 37.5% of the respondents were male and mean BMI was 25.1 kg/m^2^. 

Poisson regression models of the association between chance of a low protein intake (Pro55+) and behavioral determinants are presented in Table 3. Model 0 showed that lower scores for each determinant were associated with a higher chance of a low protein intake. Compared to the first quartile (Q1), especially the third (Q3) and fourth quartile (Q4) showed decreased prevalence ratios (PR) for most determinants on the chance of a low protein intake. “Perceived cues” was the only behavioral determinant not significant for Q3, but other effect sizes ranged between 0.63 (“self-efficacy”) and 0.77 (“social support”). For Q4, effect sizes differed between 0.49 (“intention”) and 0.70 (“perceived cues”). Effect sizes attenuated after adjustment for age, gender, BMI, living situation and income (model 1), but the trend towards a protective effect remained. When all behavioral determinants were included in the final model (model 2), prevalence ratios changed drastically. The protective effect ceased, and most PRs were no longer significant, except for “knowledge” (PR Q4 = 0.71; 95% C.I. = 0.52–0.96) and “social support” (PR Q4 = 0.71; 95% C.I. = 0.52–0.96). For all non-significant behavioral determinants, the pattern of a decreasing PR over the quartiles remained intact “for attitude, intention” and “self-efficacy”, but no longer for “cognizance, perceived cues” and “risk perception”.

## 4. Discussion

This study aimed to explore the chance of a low protein intake regarding eight behavioral determinants of the I-Change model among Dutch community-dwelling older adults. In short, almost 40% of respondents had a high chance of a low protein intake (<1 g/kg bw/day). Overall, respondents with lower scores for each of the behavioral determinants had a higher chance of a low protein intake. This effect remained when adjusting for age, gender, BMI, living situation and income. The full model, in which all behavioral determinants were included, showed that “knowledge and social support” had an independent association with the chance of a low protein intake. 

The corresponding phases in the I-Change model are awareness for “knowledge” and motivation for “social support”. Several previous studies have reported associations between nutrition-related knowledge and dietary behavior or nutrition-related knowledge and health status among (older) adults [26,27,28,29], although not specifically aimed at protein intake. A study by Jeruszka-Bielak et al. (2018) showed that good nutrition-related knowledge was associated with a lower BMI and higher physical activity in a large European cohort of older adults [26]. Studies by Spronk et al. (2021) and De Vriendt et al. (2009) showed that higher nutrition-related knowledge resulted in better dietary behavior, mostly a higher intake of fruits and vegetables [27,28]. Even though protein intake was not taken into account in these studies and effects of good nutrition-related knowledge often related to a higher intake of fruits and vegetables, these studies and ours, underline the importance of nutrition-related knowledge on eating behavior and lifestyle. Kok et al. (2016) also regarded “knowledge” to be the basis for many other determinants [13]. Similar to the results of our study, the authors described that most behavioral determinants are not independent of each other. In practice, this may imply that interventions that aim to increase protein intake among Dutch community-dwelling older adults should focus on improvement in knowledge as the basis for multiple behavioral determinants and different stages in the I-Change model. Even though the latter studies were not specifically aimed at increasing protein intake, a recent study by Yung Hung et al. (2019) also addressed different behavioral determinants in relation to protein intake among Dutch older adults [29]. Similar to our study, “knowledge” was low in participants with a high chance of a low protein intake. In addition, difficulties in meal preparation (“self-efficacy”), the ability to engage in physical activities in difficult situations (“self-efficacy”) and a lower readiness to follow dietary advice (“attitude/intention”) were found to be associated with a lower protein intake. Similarities between this study and the current study are that behavioral determinants of the motivation phase explain a low protein intake among Dutch community-dwelling older adults and that “knowledge” is an important behavioral determinant within the awareness phase. Differences can be explained by the different methods used to determine the different behavioral determinants. 

Previous studies also revealed that “social support” has a large influence on dietary behavior, but again these studies in both young (age 17–47 y) and older adults (>50 y) were specifically aimed at fruit and vegetable intake [30,31,32]. These studies reported either a synergistic or adverse effect between social support and self-efficacy related to action plans (in I-Change model: action phase) or actual behavior. Our study indicates that social support has an independent effect on dietary behavior. This difference may be because other studies focused on the intake of fruit and vegetables, rather than protein intake, and included participants in different age groups. Altogether, future interventions should target the behavioral determinants “knowledge and social support” to increase protein intake among Dutch community-dwelling older adults. 

To our knowledge, this study is one of the first to assess specific behavioral determinants related to protein intake in community-dwelling older adults. A large cohort of 824 respondents was recruited via several channels, to include a representative population of Dutch community-dwelling older adults. The mean BMI of our population (25.1 kg/m^2^) was lower compared to that in similar studies in community-dwelling older adults (~27 kg/m^2^) [11,33]. Additionally, the proportion of respondents with a high education was higher compared to the Dutch population [34]. This may imply that our population already had a healthier diet, as previous studies showed that educational level is positively associated with a healthy lifestyle and a lower BMI [35,36,37,38]. Therefore, future research should also include older adults with a low-to-middle educational level and/or a higher BMI to assess possible differences within this population. 

The items included in the questionnaire were based on existing, validated questionnaires. The questionnaire had been pre-piloted among a group of Dutch community-dwelling older adults to check for readability and comprehension. Even though we assume that the questionnaire is valid for our intended purpose, the questionnaire as a whole was not validated. Therefore, a possible limited validity of the final instrument should be acknowledged. 

The Pro55+ is a very time-efficient tool to assess protein intake. However, it is an indirect measurement, estimating the chance of a protein intake below 1.0 g/kg BW/day. The tool is not designed to accurately determine the protein intake of the respondents. For this, classical methods such as a food frequency questionnaire or food diary should be used, but these methods are time consuming and not feasible for this study. However, this screening tool has been validated in a population of Dutch community-dwelling older adults and has shown to be a good estimation of whether respondents have a protein intake below or above 1.0 g/kg BW/day with an area under the curve of 0.856 compared to a population of 5188 Dutch respondents [17]. Another limitation may be that only a few respondents chose the first three categories (totally disagree–disagree a little) of the 7-point Likert scale. Using this type of scale made it impossible to determine the relative outcome per determinant, e.g., whether respondents had a “good” or “bad” attitude towards eating enough protein per day/meal moment. It was also impossible to analyze the outcomes continuously. Therefore, outcomes were divided into quartiles to distinguish between respondents who had higher scores on the 7-point Likert scale. Finally, we did not ask for underlying diseases such as chronic kidney disease or other health issues necessitating a protein restriction and we cannot exclude that a few respondents may have followed a protein restricted diet.

## 5. Conclusions

In conclusion, this study shows that the behavioral determinants “knowledge and social support” are independently associated with the chance of a low protein intake. In practice, this means that increasing knowledge and social support may improve the protein intake of Dutch community-dwelling older adults and thus ensure that a larger proportion of this population meets protein recommendations. Even though increasing knowledge sounds like a relatively simple solution, in practice this might be quite challenging. Older adults tend to have specific preferences for the type of communication strategy, e.g., storytelling is a strategy that attracts older adults [39,40]. Additionally, the messenger is important to older adults: formal support, for instance by a GP, can help in a successful transition of knowledge. However, the group of older adults is heterogenous, e.g., in educational level, thus different groups might need different approaches. Hence, future studies should include older adults with a lower educational level and a higher BMI, in order to provide insights into possible differences between these groups. Additionally, intervention studies need to be developed that are tailored to the needs of the heterogenous group of Dutch community-dwelling older adults.

## Figures and Tables

**Table 1:** Items in the questionnaire, presented per determinant of behavior and time of day.

Knowledge		
In general	In your opinion, which foods in the figures below contain dietary protein? Twelve food products were shown	
Attitude ^◊^		Cronbach α = 0.88
During the day	Consuming enough protein-rich foods, spread throughout the day, is important to me. Consuming enough protein-rich foods, spread throughout the day, is healthy. Consuming enough protein-rich foods, spread throughout the day, is desirable.	
During breakfast	Consuming enough protein-rich foods, during breakfast, is important to me. Consuming enough protein-rich foods, during breakfast, is healthy. Consuming enough protein-rich foods, during breakfast, is desirable.	
Risk perception ^◊^		Cronbach α = 0.75
During the day	A low intake of dietary protein during the day has negative consequences for my health status. When I don’t consume enough protein-rich foods during the day, physical exercise becomes more difficult. When I don’t consume enough protein-rich foods during the day, I feel more tired.	
Cognizance ^◊^		Cronbach α = 0.55
During the day	I think I eat enough protein-rich foods during the day. *	
During breakfast	I think I eat enough protein-rich foods during breakfast. *	
Self-efficacy ^◊^		Cronbach α = 0.63
During the day	I can eat enough protein-rich foods during the day. *	
During breakfast	I can eat enough protein-rich foods during breakfast. *	
Perceived cues ^◊^		Cronbach α = 0.69
During the day	No one has ever told me that eating enough protein during the day is important for my health status. I know from people around me who had to eat more dietary protein due to disease, that a sufficient intake of protein during the day is important for good health.	
During breakfast	No one has ever told me that eating enough protein during breakfast is important for my health status. I know from people around me who had to eat more dietary protein due to disease, that a sufficient intake of protein during breakfast is important for good health.	
Social support ^◊^		Cronbach α = 0.81
During the day	People that are close to me eat enough dietary protein during the day. * People that are close to me motivate/support me to eat enough dietary protein during the day. *	
During breakfast	People that are close to me eat enough dietary protein during the day. * People that are close to me motivate/support me to eat enough dietary protein during the day. *	
Intention ^◊^		Cronbach α = 0.75
During the day	I plan to eat enough dietary protein throughout the day for the upcoming months. *	
During breakfast	I plan to eat enough dietary protein during breakfast for the upcoming months. *	

* Examples of protein-rich foods were shown below the question; ^◊^ 7-point scale.

**Table 2:** Sociodemographic characteristics of all respondents and for respondents with a high and low chance of a low protein intake.

	Total	Protein Screener ≤ 0.3 Low Chance of Low Protein Intake *	Protein Screener > 0.3 High Chance of Low Protein Intake
	824	499 (60.6%)	325 (39.4%)
Age			
Mean (±SD)	72.9 (5.9)	72.6 (5.8)	73.5 (6.1)
65–74	518	328 (65.7%)	190 (58.4%)
75–84	264	149 (29.9%)	115 (35.4%)
≥85	42	22 (4.4%)	20 (6.2%)
Sex			
Male	309 (37.5%)	167 (33.5%)	142 (43.7%)
Female	515 (62.5%)	332 (66.5%)	183 (56.3%)
BMI (kg/m^2^)			
Mean (±SD)	25.1 (3.7)	24.6 (4.0)	25.9 (3.2)
<20	44	37 (7.4%)	7 (2.2%)
20–27	567	353 (70.7%)	215 (66.1%)
>27	212	109 (21.8%)	103 (31.7%)
Living situation			
Living alone	310	186 (37.3%)	124 (38.2%)
Living together **	514	313 (62.7%)	201 (61.8%)
Living area			
Urban	394	218 (43.7%)	176 (54.2%)
Suburban	379	249 (49.9%)	130 (40%)
Rural	51	32 (6.4%)	19 (5.8%)
Education			
Low	228	140 (28.1%)	88 (27.1%)
Middle	202	120 (24.0%)	82 (25.2%)
High	394	239 (47.9%)	155 (47.7%)
Income ***			
Low	170	91 (18.2%)	79 (24.3%)
High	654	408 (81.8%)	246 (75.7%)

* Protein intake was estimated with Pro55+, with low protein intake defined as <1.0 g/kg BW/day. ** With partner and/or children. *** Low income was defined as annual income <€30.481 for singles and <€38.945 for couples.

**Table 3:** Association between eight behavioral determinants and outcomes of the Pro55+ outcomes in bold are considered significant.

	Quartile (Median (Range))	N	Model 0: Prevalence Ratio (95% C.I.)	Model 1 *: Adjusted Prevalence Ratio (95% C.I.)	Model 2 **: Full Model (95% C.I.)
Attitude	Q1 (4.5 (1.5–5.0))	222	Ref	Ref	Ref
	Q2 (5.5 (5.2–5.7))	195	1.00 (0.81–1.22)	1.02 (0.83–1.25)	1.12 (0.90–1.39)
	Q3 (6.0 (5.8–6.0))	192	**0.75** **(0.59–0.95)**	0.79 (0.63–1.00)	1.03 (0.77–1.36)
	Q4 (6.5 (6.2–7.0))	215	**0.59** **(0.46–0.76)**	**0.62** **(0.48–0.80)**	0.93 (0.66–1.31)
Cognizance	Q1 (4.0 (1.0–4.5))	196	Ref	Ref	Ref
	Q2 (5.5 (5.0–5.5))	228	0.84 (0.69–1.03)	0.83 (0.68–1.01)	0.93 (0.73–1.19)
	Q3 (6.0 (6.0–6.0))	256	**0.64** **(0.51–0.80)**	**0.66** **(0.53–0.83)**	0.91 (0.67–1.23)
	Q4 (7.0 (6.5–7.0))	144	**0.60** **(0.45–0.79)**	**0.63** **(0.48–0.84)**	1.30 (0.85–1.99)
Intention	Q1 (4.0 (1.0–4.5))	179	Ref	Ref	Ref
	Q2 (5.0 (5.0–5.5))	159	0.95 (0.77–1.17)	0.95 (0.77–1.17)	0.96 (0.78–1.20)
	Q3 (6.0 (6.0–6.0))	273	**0.68** **(0.55–0.84)**	**0.70** **(0.57–0.86)**	0.84 (0.65–1.09)
	Q4 (7.0 (6.5–7.0))	213	**0.49** **(0.38–0.64)**	**0.51** **(0.39–0.67)**	0.70 (0.48–1.00)
Knowledge	Q1 (1.0 (−4.0–2.0))	189	Ref	Ref	Ref
	Q2 (4.0 (3.0–4.0))	285	0.98 (0.79–1.20)	1.03 (0.84–1.27)	1.01 (0.83–1.23)
	Q3 (5.0 (5.0–5.0))	230	**0.76** **(0.60–0.97)**	0.83 (0.65–1.06)	0.81 (0.64–1.02)
	Q4 (6.0 (6.0–6.0))	120	**0.67** **(0.49–0.92)**	**0.74** **(0.54–1.01)**	**0.71** **(0.52–0.97)**
Perceived cues	Q1 (3.0 (1.0–3.5))	230	Ref	Ref	Ref
	Q2 (4.0 (3.8–4.0))	192	1.02 (0.81–1.27)	0.98 (0.79–1.22)	1.15 (0.92–1.43)
	Q3 (4.8 (4.3–5.0))	187	1.0 (0.80–1.26)	0.98 (0.78–1.22)	1.19 (0.94–1.47)
	Q4 (6.0 (5.3–7.0))	215	**0.70** **(0.54–0.90)**	**0.71** **(0.55–0.91)**	0.97 (0.74–1.26)
Risk perception	Q1 (4.0 (1.3–4.3))	166	Ref	Ref	Ref
	Q2 (5.0 (4.7–5.3))	283	0.93 (0.76–1.14)	0.99 (0.81–1.21)	1.04 (0.84–1.30)
	Q3 (5.7 (5.7–5.7))	99	**0.70** **(0.51–0.97)**	0.76 (0.55–1.06)	0.89 (0.64–1.25)
	Q4 (6.0 (6.0–7.0))	276	**0.67** **(0.53–0.85)**	**0.71** **(0.56–0.89)**	1.00 (0.75–1.33)
Self-efficacy	Q1 (4.0 (1.0–4.5))	167	Ref	Ref	Ref
	Q2 (5.5 (5.0–5.5))	209	0.93 (0.76–1.14)	0.95 (0.77–1.16)	1.06 (0.84–1.35)
	Q3 (6.0 (6.0–6.0))	282	**0.63** **(0.51–0.79)**	**0.65** **(0.52–0.81)**	0.85 (0.63–1.15)
	Q4 (7.0 (6.5–7.0))	166	**0.51** **(0.38–0.68)**	**0.53** **(0.40–0.71)**	0.64 (0.41–1.01)
Social support	Q1 (2.5 (1.0–3.0))	199	Ref	Ref	Ref
	Q2 (4.0 (3.3–4.0))	271	0.88 (0.72–1.07)	0.88 (0.72–1.06)	0.83 (0.68–1.02)
	Q3 (4.5 (4.3–5.0))	180	**0.77** **(0.61–0.98)**	**0.76** **(0.60–0.96)**	0.82 (0.64–1.04)
	Q4 (6.0 (5.3–7.0))	174	**0.56** **(0.42–0.74)**	**0.54** **(0.41–0.72)**	**0.71** **(0.52–0.96)**

All PRs (with 95% C.I.) are based on Poisson regression analyses. PRs and CIs in bold are significant. * Adjusted for age (65–74 y; 75–84 y; >85 y), gender (male/female), BMI (<20 kg/m^2^; 20–27 kg/m^2^; >27 kg/m^2^), living situation (alone/together) and income (low/high). ** Model included all behavioral determinants (cognizance, knowledge, risk perception, perceived cues, attitude, social support, self-efficacy and intention). All behavioral determinants were based on outcomes on 7-point scale except for knowledge, which was based on a scoring system (ranging from −6–+6).

## Data Availability

The data presented in this study are available on request from the corresponding author. The data are not publicly available due to privacy restrictions.

## Appendix A

**Table A1:** Association between cognizance and self-efficacy and outcomes of Pro55+, stratified per meal moment. PRs and CIs in bold are significant.

	Quartile (Median (IQR))	N	Prevalence Ratio (95% C.I.)
Cognizance	Q1 (5.0 (1.0–5.0))	255	Ref
During breakfast	Q2 (6.0 (6.0–6.0))	428	**0.54** **(0.41–0.73)**
	Q3 (7.0 (7.0–7.0))	141	**0.68** **(0.57–0.81)**
Cognizance	Q1 (3.0 (1.0–4.0))	198	Ref
During the day	Q2 (5.0 (5.0–5.0))	179	0.95 (0.77–1.18)
	Q3 (6.0 (6.0–6.0))	315	**0.72** **(0.58–0.88)**
	Q4 (7.0 (7.0–7.0))	132	**0.63** **(0.47–0.85)**
Self-efficacy	Q1 (4.0 (1.0–50))	257	Ref
During breakfast	Q2 (6.0 (6.0–6.0))	406	**0.69** **(0.58–0.82)**
	Q3 (7.0 (6.0–7.0))	161	**0.51** **(0.38–0.67)**
Self-efficacy	Q1 (4.0 (1.0–4.0))	173	Ref
During the day	Q2 (5.0 (5.0–5.0))	158	1.07 (0.87–1.32)
	Q3 (6.0 (6.0–6.0))	333	**0.65** **(0.53–0.81)**
	Q4 (7.0 (7.0–7.0))	160	**0.55** **(0.41–0.73)**
